# Role of IL-10-producing regulatory B cells in modulating T-helper cell immune responses during silica-induced lung inflammation and fibrosis

**DOI:** 10.1038/srep28911

**Published:** 2016-06-29

**Authors:** Fangwei Liu, Wujing Dai, Chao Li, Xiaowei Lu, Ying Chen, Dong Weng, Jie Chen

**Affiliations:** 1 Division of Pneumoconiosis, School of Public Health, China Medical University, Shenyang, P. R. China; 2 Department of Respiratory Medicine, Shanghai Pulmonary Hospital, Tongji University School of Medicine, Shanghai, P. R. China

## Abstract

Silicosis is characterized by chronic lung inflammation and fibrosis, which are seriously harmful to human health. Previous research demonstrated that uncontrolled T-helper (Th) cell immune responses were involved in the pathogenesis of silicosis. Lymphocytes also are reported to have important roles. Existing studies on lymphocyte regulation of Th immune responses were limited to T cells, such as the regulatory T (Treg) cell, which could negatively regulate inflammation and promote the process of silicosis. However, other regulatory subsets in silicosis have not been investigated in detail, and the mechanism of immune homeostasis modulation needs further exploration. Another regulatory lymphocyte, the regulatory B cell, has recently drawn increasing attention. In this study, we comprehensively showed the role of IL-10-producing regulatory B cell (B10) in a silicosis model of mice. B10 was inducible by silica instillation. Insufficient B10 amplified inflammation and attenuated lung fibrosis by promoting the Th1 immune response. Insufficient B10 clearly inhibited Treg and decreased the level of IL-10. Our study indicated that B10 could control lung inflammation and exacerbate lung fibrosis by inhibiting Th1 response and modulating the Th balance. The regulatory function of B10 could be associated with Treg induction and IL-10 secretion.

Inhalation of silica particles can induce silicosis, which is characterized by chronic lung inflammation and irreversible fibrosis. Although many governments strive to prevent exposure to silica, the global incidence of silicosis is still unacceptably high. There also is an increasing trend toward younger populations developing silicosis, which results in a heavy burden to human health and national health care systems^1^. Silica instillation induces alveolar cell injury, followed by the interstitial infiltration of numerous inflammatory cells. Then, fibroblast proliferation and extracellular matrix deposition repeatedly occur in the lung, which leads to lung fibrosis. The regulatory mechanism of silica-induced lung inflammation and fibrosis still needs further study.

Previous evidence supports both innate and adaptive immune responses in the pathogenesis of silicosis. Many immune cells are involved in the uncontrolled immune process of silicosis. Lymphocytes are reported to have crucial roles in the development of silica-induced lung inflammation and fibrosis, especially CD4^+^ T cells^2^. After silica particle recognition by macrophages, naïve T cells could be activated through interaction with antigen-presenting cells. Multiple CD4^+^ T cells, including Th1, Th2, and Th17, participate in the immune response. The Th1 response is dominant during the early inflammatory stage^2^,^3^. Whereas, the Th2 response is elevated during the development of late fibrosis. The Th17 response also is involved in the initial stage of silicosis. IL-17 neutralization can influence the traditional Th1/Th2 immune balance after silica instillation^4^,^5^,^6^. We previously demonstrated that CD4^+^Foxp3^+^ Treg was involved in modulation of Th immune balance after silica instillation. Depletion of Treg clearly reduced the level of IL-10, which could positively regulate the process of silica-induced lung inflammation and fibrosis^2^,^7^.

Recent studies indicate that a novel regulatory subset of CD19^+^ B lymphocytes is involved in controlling inflammation, autoimmune disease, allergic disease, and tumorigenesis^8^,^9^,^10^,^11^. CD19^−/−^ mice show lower susceptibility to bleomycin challenge, and CD19 overexpression aggravates lung fibrosis^12^. CD19 depletion has been shown to dramatically enhance the T-cell-mediated inflammatory response^13^. CD19^−/−^ mice have a dramatic reduction in the number of B cells in peripheral lymphoid tissues and the number of regulatory B cells^13^,^14^. CD19^+^ regulatory B cells exhibit various phenotypes, including CD1d^hi^CD5^+^, CD24^hi^CD38^hi^, and CD21^+^CD23^+^ 15,16. IL-10 secretion is the characteristic activity of regulatory B cells in regulating inflammatory disease; therefore, CD19^+^ and IL-10^+^ are commonly used as markers for IL-10-producing regulatory B cells (B10)^17^,^18^. B10 participates in modulating Th immune response by affecting the secretion of inflammatory cytokines such as TNF-α, IFN-γ, IL-12, and IL-17 19,20. The relationship between B10 and other regulatory cells is discussed in several recent studies^21^,^22^,^23^. The current hypothesis is that B10 might influence the proliferation of CD4^+^ T cells including Treg^24^,^25^. The role of IL-10 in B10 regulation is still subject to debate^25^,^26^. Whether B10 regulatory function relies on Treg and/or IL-10 in silica-induced lung inflammation and fibrosis is unknown. The mechanism of B10 in regulating immune homeostasis is still poorly interpreted in silicosis.

In this study, we investigated the role of B10 during silica-induced lung inflammation and fibrosis. We examined the regulatory function of B10 on Th immune response, and the reciprocal relationship between B10 and Treg in silicosis. We found that insufficient B10 amplified the inflammation and attenuated lung fibrosis, mainly via modulating the Th balance. Insufficient B10 could shift the Th balance toward Th1-predominance by modulating Treg and inhibitory cytokine IL-10.

## Results

### B10 is involved in the development of silica-induced lung inflammation and fibrosis

**Figure 1:**
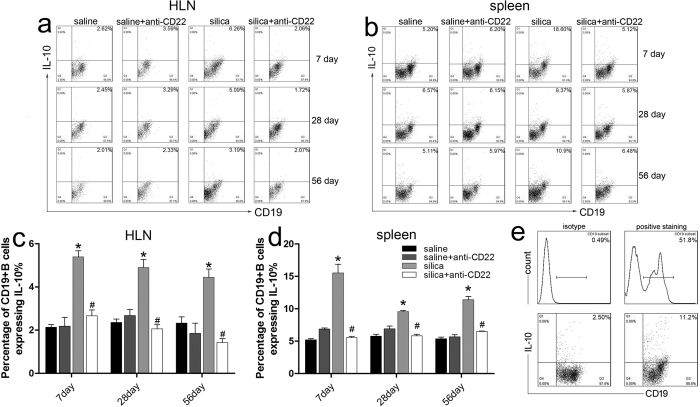
Anti-CD22 attenuates B10 induction *in vivo* after silica instillation. (**a**,**c**) C57BL/6 mice were treated i.p. with 300 μg anti-CD22 monoclonal antibody or control IgG, and the percentage of CD19^+^ IL-10^+^ regulatory B cells (B10) in the hilar lymph node was assayed by flow cytometry. (**b**,**d**) Percentage of B10 in spleen is shown in the graph. (**e**) CD19 positive gate and IL-10 positive gate were drawn according to their isotype control staining. (*n* = 5; *P < 0.05 compared with the saline control group; ^#^*P* < 0.05 compared with the silica group).

The initial experiment described here addressed whether B10 was involved in the development of silica-induced lung inflammation and fibrosis. Figure 1 shows that the percentage of CD19^+^IL-10^+^ B cells in the hilar lymph node (HLN) increased dramatically after silica instillation and then gradually declined. The CD19^+^IL-10^+^ B cell fraction in silica-treated mice remained higher than that in the saline-treated (control group) mice (Fig. 1a,c). Similar results were observed in spleen; the percentage of CD19^+^IL-10^+^ B cells in the silica-treated group increased significantly compared with the control group on day 7, 28, and 56 after silica instillation (Fig. 1b,d). All the gates were drawn according to the isotype staining (Fig. 1e). These data suggest that B10 is involved in the development of silica-induced lung inflammation and fibrosis. Next, to further identify its role, a B10-deficiency mouse model was generated by intraperitoneal injection of anti-CD22. The efficacy of anti-CD22 was assessed by flow cytometry. Compared with the silica-treated group, mice treated with anti-CD22 exhibited lower levels of B10 in both HLN and spleen after silica instillation, which indicates that anti-CD22 treatment attenuates silica-induced B10 induction (Fig. 1).

### Insufficient B10 exacerbates the inflammatory response induced by silica

**Figure 2:**
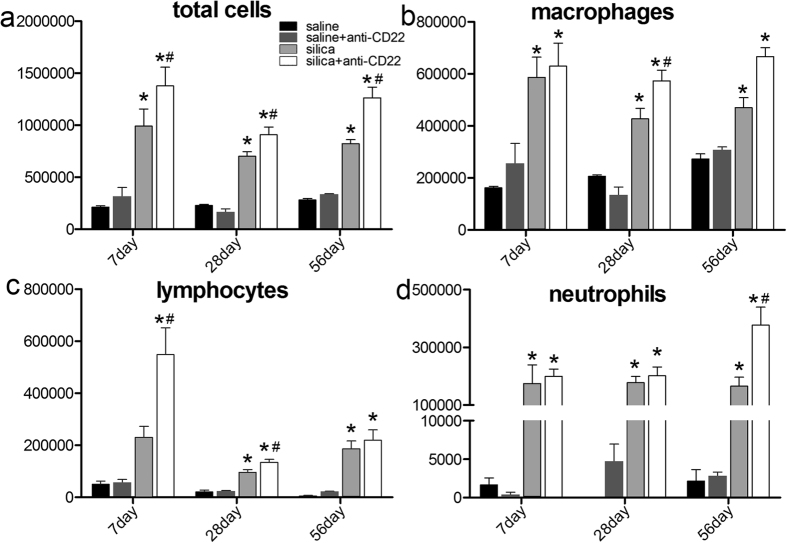
Insufficient B10 enhances accumulation of inflammatory cells in lung after silica instillation. (**a**) Total cells, (**b**) macrophages, (**c**) lymphocytes, and (**d**) neutrophils in BALF were counted using Giemsa staining (*n* = 5; *P < 0.05 compared with the saline control group; ^#^*P* < 0.05 compared with the silica group).

Inhalation of silica particles induces lung inflammation, characterized by the accumulation of inflammatory cells, pathological changes in lung, and secretion of pro-inflammatory cytokines. To examine the influence of B10 on silica-induced lung inflammation, we counted the number of inflammatory cells in bronchoalveolar lavage fluid (BALF) after Giemsa staining. It was visually apparent that a large number of inflammatory cells were accumulated in BALF on day 7, 28, and 56 after silica instillation. The total number of cells in the group treated with silica and anti-CD22 was considerably higher than that in the group treated only with silica on day 7 and 56 after treatment (Fig. 2a). Different cell types contributed to this change at different stages of silica-induced lung inflammation. After anti-CD22 treatment, mice showed obvious enhancement of macrophages on day 28 and 56 (Fig. 2b). The levels of lymphocytes in the group treated with silica and anti-CD22 increased substantially on day 7 compared with that in the group treated only with silica, and then declined to comparable levels as those in the silica-treated group on day 28 and 56 (Fig. 2c). The B10 effect on neutrophil recruitment appeared primarily at a late stage (Fig. 2d). These results suggest that B10 insufficiency leads to the accumulation of much more inflammatory cells in BALF after silica instillation, primarily early lymphocytes, late macrophages, and neutrophils.

**Figure 3:**
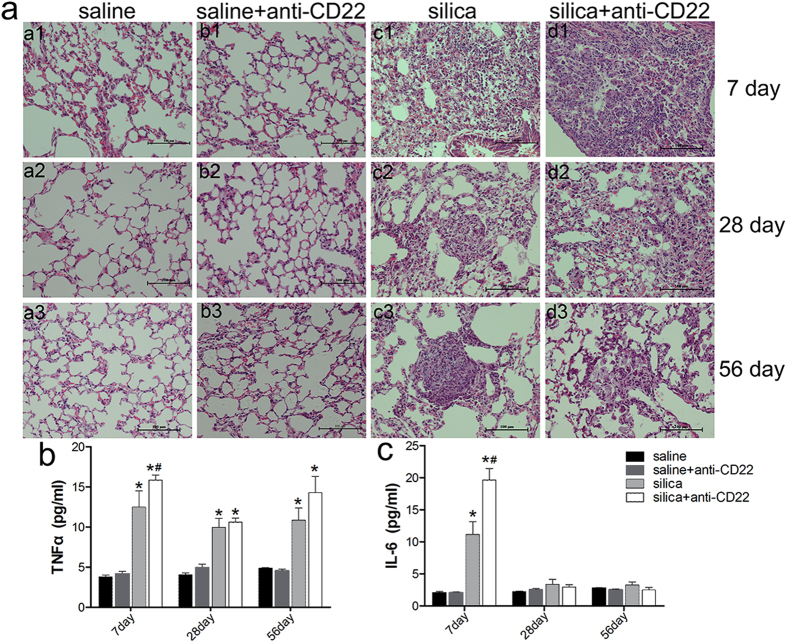
Insufficient B10 exacerbates the inflammatory response after silica instillation. (**a**) Histopathology changes in mouse lungs after silica instillation observed with H&E staining (×200). a1–d1, day 7; a2–d2, day 28, and a3–d3, day 56. a1–a3, saline group; b1–b3, saline plus anti-CD22 group; c1–c3, silica group; d1–d3, silica plus anti-CD22 group. (**b**) Secretion of pro-inflammatory cytokine TNF-α in BALF was evaluated by CBA analysis. (**c**) Secretion of IL-6 in BALF was assayed by CBA analysis (*n* = 5; *P < 0.05 compared with the saline control group; ^#^*P* < 0.05 compared with the silica group).

To investigate the impact of B10 on the pathogenesis of silica-induced lung inflammation, mouse lung section was observed by light microscopy after staining with hematoxylin and eosin (H&E) to monitor pathological changes. No obvious abnormalities were observed in the saline-treated control group and the group treated with saline and anti-CD22, which indicated that the antibody did not induce obvious pathological changes in lung (Fig. 3a). Mice in the silica-treated group had a large infiltration of inflammatory cells, and exhibited alveolar septal thickening and cellular nodules (grade I) on day 7 after treatment. Severe infiltrations of inflammatory cells and more irregular cellular nodules were observed in the group treated with silica and anti-CD22 on day 7 after treatment. Mice in the silica-treated showed considerably reduced inflammation on day 28, along with the appearance of large fibrotic cellular nodules (grade II). However, the remission of inflammation was not observed in the group treated with silica and anti-CD22 on day 28. On 56 days after treatment, mice in the silica-treated group exhibited increased levels of fibrotic cellular nodules (grade II). Cellular fibrotic nodules (grade III) also appeared in the silica-treated group. In the group treated with silica and anti-CD22, irregular cellular nodules and fibrotic cellular nodules were observed on day 56. The grade of silicotic nodules was estimated according to the criteria described in Methods. The grade of silicotic nodules in the silica plus anti-CD22 group was lower than that in silica group (see Supplementary Table S1). These data indicate that insufficient B10 exacerbates the inflammatory pathology changes and alleviates the development of silicotic nodules.

To further evaluate the inflammatory changes in lung, the representative pro-inflammatory cytokines TNF-α and IL-6 were examined by cytometric beads array (CBA). Figure 3b shows that TNF-α secretion clearly increased after silica instillation at all three time points (day 7, 28, and 56). The induction of TNF-α was further amplified by anti-CD22 application. The difference between the silica plus anti-CD22 group and the silica group was significant especially on day 7. Silica instillation also elevated IL-6 secretion on day 7 (Fig. 3c). The secretion of IL-6 was significantly higher in the silica plus anti-CD22 group than in the silica group during the early time point. These combined results indicate that insufficient B10 could amplify lung inflammation after silica instillation.

### Insufficient B10 attenuates silica-induced lung fibrosis

**Figure 4:**
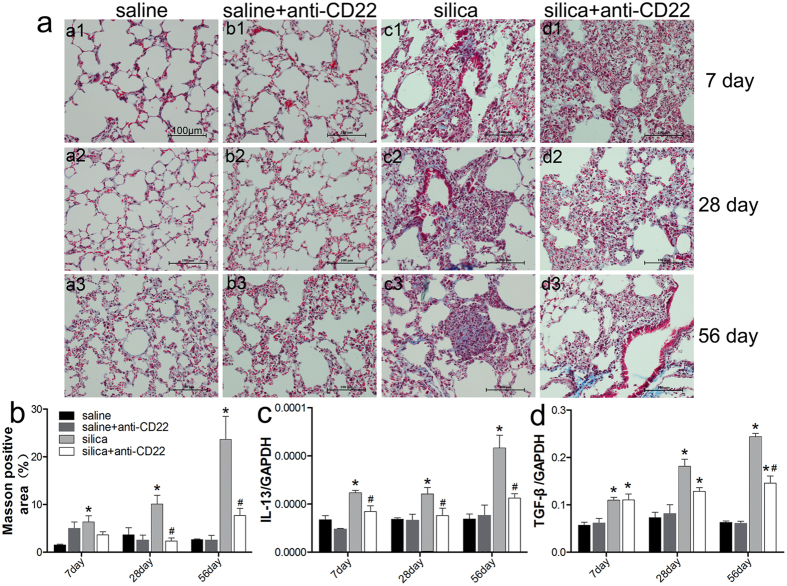
Insufficient B10 attenuates the fibrotic response during silica-induced lung fibrosis. (**a**) Lung sections from C57BL/6 mice were stained with Masson trichrome stain and observed under the light microscope (×200). a1–d1, day 7; a2–d2, day 28, and a3–d3, day 56. a1–a3, saline group; b1–b3, saline plus anti-CD22 group; c1–c3, silica group; d1–d3, silica plus anti-CD22 group. (**b**) The area of positive collagen deposition in lung sections was visualized by Masson staining and quantified using image-pro plus. (**c**) Relative IL-13 expression in lung was assayed by real time PCR. (**d**) Relative expression of TGF-β in lung was assayed by real time PCR (*n* = 5; *P < 0.05 compared with the saline control group; ^#^*P* < 0.05 compared with the silica group).

Treatment with anti-CD22 influenced the development of cellular nodules as observed by H&E staining. To confirm these results, Masson staining and real time PCR analyses were used to study collagen deposition and pro-fibrotic response. Masson staining revealed that silica instillation induced lung collagen deposition (Fig. 4a). In the silica group, collagen deposition increased on day 28 and 56 after treatment, along with the development of fibrotic cellular nodules. Collagen deposition in the lung section was quantified according to Masson staining. The results indicated that lung collagen deposition was limited in the silica plus anti-CD22 group (7.67%) compared with that in the silica group (23.65%) on day 56 after silica instillation (Fig. 4b). Changes in collagen deposition were associated with differences in nodule development in these two groups. These results suggest that insufficient B10 could restrict collagen deposition after silica instillation.

Next, expressions of the fibrosis-related genes IL-13 and TGF-β during silica-induced lung fibrosis were evaluated by real time PCR analysis. The expression of IL-13 increased and reached a peak on day 56 after silica instillation, whereas the expression of IL-13 in the silica plus anti-CD22 group was consistently lower than that in the silica group at all three time points (Fig. 4c). The accumulated literature reports that TGF-β has a crucial role in tissue fibrosis. In the current study, the expression of TGF-β increased gradually after silica instillation (Fig. 4d). Treatment with anti-CD22 significantly attenuated silica-induced TGF-β expression on day 56 (Fig. 4d). These combined data indicate that insufficient B10 attenuates the pro-fibrotic response.

### B10 regulation of silica-induced lung inflammation and fibrosis could be associated with modulation of Th immune balance

Previous studies show that the Th immune response plays a major role in the development of silica-induced lung inflammation and fibrosis^27^,^28^,^29^. Our preceding study reported that the level of Th1 cytokines increased during the early stage of silicosis, whereas the level of Th2 cytokines increased during the late stage of silicosis, which indicates that the Th balance shifts from Th1 predominant to Th2 predominant during fibrosis development^3^,^7^. Th17 also was involved in silica-induced lung inflammation and fibrosis. To further evaluate whether the changes in inflammatory responses resulting from insufficient B10 were related to Th responses and Th balance, we performed flow cytometry, real time PCR, and CBA analyses.

**Figure 5:**
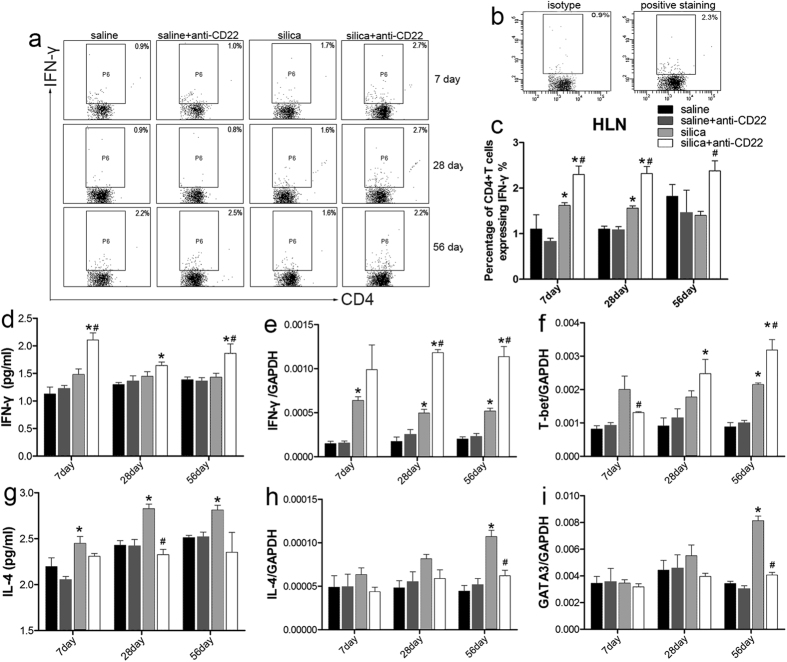
Insufficient B10 affected the Th1/Th2 balance in the mouse model of silica-induced lung fibrosis. (**a**,**c**) Percentage of CD4^+^ IFN-γ^+^ Th1 cells in the hilar lymph node was assayed by flow cytometry. (**b**) IFN-γ positive gate were drawn according to their isotype control staining. (**d**) Secretion of typical Th1 cytokine IFN-γ in BALF was assayed by CBA. (**e**,**f**) Relative expression levels of typical Th1 cytokine IFN-γ and Th1 nuclear transcription factor T-bet in lung were assayed by real time PCR. (**g**) Secretion of typical Th2 cytokine IL-4 in BALF was assayed by CBA. (**h**,**i**) Relative expression levels of typical Th2 cytokine IL-4 and Th2 nuclear transcription factor GATA3 in lung were assayed by real time PCR (*n* = 5; *P < 0.05 compared with the saline control group; ^#^*P* < 0.05 compared with the silica group).

The percentage of CD4^+^ T cells expressing IFN-γ, identified as Th1 cells, significantly increased in the silica plus anti-CD22 group compared with that of the silica group (Fig. 5a,c). IFN-γ positive gate was drawn according to the isotype staining (Fig. 5b). Anti-CD22 treatment amplified IFN-γ expression and secretion after silica instillation (Fig. 5d,e). A similar result was observed in the expression of the Th1 transcription factor T-bet on day 28 and 56 (Fig. 5f). The Th1 response was exacerbated by insufficient B10. The Th1/Th2 balance is well known for many diseases, including inflammation, cancer, and autoimmune disease. In the current study, the expression and secretion of the Th2 cytokine IL-4 were considerably reduced during the late stage in the silica plus anti-CD22 group compared with that in the silica group (Fig. 5g,h). The level of the Th2 transcription factor GATA3 after silica instillation was reduced by anti-CD22 application on day 56 (Fig. 5i). These results suggest that the Th2 response was attenuated by anti-CD22 application after silica instillation.

**Figure 6:**
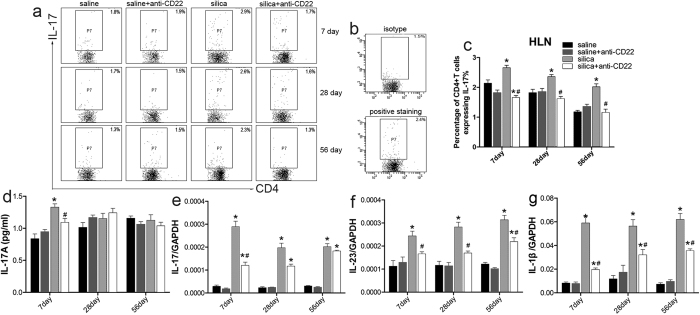
Insufficient B10 regulates the Th17 immune response during silica-induced lung inflammation and fibrosis. (**a**,**c**) Percentage of CD4^+^ IL-17^+^ Th17 cells in the hilar lymph node was assayed by flow cytometry. (**b**) IL-17 positive gate were drawn according to their isotype control staining. (**d**) Secretion of typical Th17 cytokine IL-17 in BALF was assayed by CBA. (**e**) Relative expression levels of typical Th17 cytokine IL-17 in lung were assayed by real time PCR. (**f**,**g**) Relative expression levels of Th17 related cytokines IL-23 and IL-1β in lung were assayed by real time PCR (*n* = 5; *P < 0.05 compared with the saline control group; ^#^*P* < 0.05 compared with the silica group).

The Th17 response was reported to be associated with the Th1 response in inflammatory disease^30^. Anti-CD22 treatment reduced the percentage of IL-17A-expressing CD4^+^ T cells, or Th17 cells, after silica instillation (Fig. 6a,c). IL-17A positive gate was drawn according to the isotype staining (Fig. 6b). The secretion and expression of typical Th17 cytokine IL-17 decreased on day 7 in the silica plus anti-CD22 group compared with that of the silica group (Fig. 6d,e). The expression of IL-23, which promoted Th17 cell differentiation, decreased in the silica plus anti-CD22 group compared with that of the silica group after silica instillation (Fig. 6f). Furthermore, the expression of IL-1β, which helps to maintain Th17 cell homeostasis, was examined by performing real time PCR analysis^31^. The results showed that the expression of IL-1β was consistently suppressed by anti-CD22 treatment after silica instillation (Fig. 6g). These data suggest that the Th17 response is suppressed by anti-CD22. The combined results indicate that insufficient B10 modulated the Th balance and maintained a dominant Th1 response.

### Treg may be involved in B10 regulation of Th responses during silica-induced lung inflammation and fibrosis

**Figure 7:**
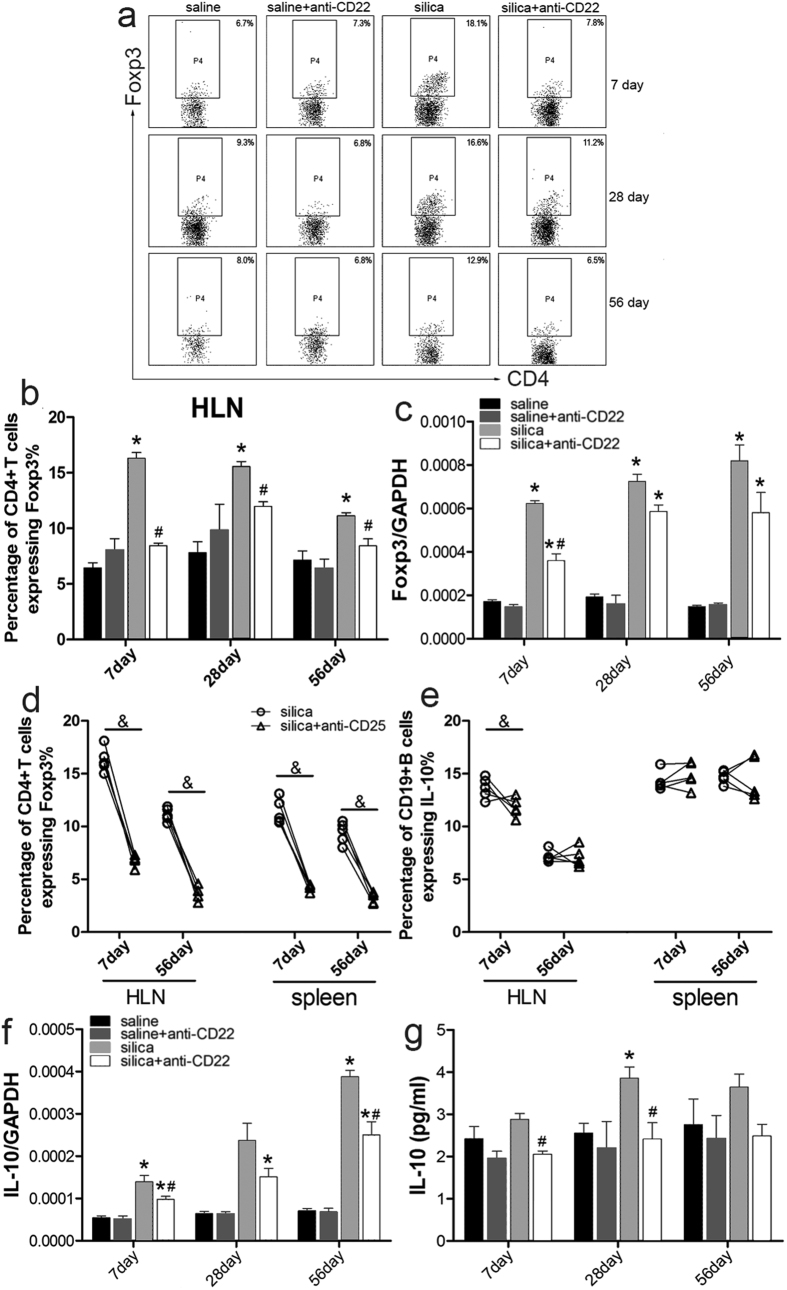
Regulatory T cells are involved in the B10 regulatory mechanism that modulates Th immune responses after silica instillation. (**a**,**b**) Percentage of CD4^+^ Foxp3^+^ Treg in the hilar lymph node was assayed by flow cytometry. (**c**) Relative expression of Treg nuclear transcription factor (Foxp3) in lung was assayed by real time PCR. (**d**) To check the efficacy of anti-CD25 in depletion of Treg, the percentage of CD4^+^Foxp3^+^Treg in hilar lymph node and spleen was assayed by flow cytometry. (**e**) Flow cytometry analysis of B10 percentage differences in hilar lymph node and spleen in Treg-depleted mice and wild-type mice after silica instillation. (**f**,**g**) Secretion and relative expression of inhibitory cytokine IL-10 in BALF and lung were assayed by CBA and real time PCR (*n* = 5; *P < 0.05 compared with the saline control group; ^#^*P* < 0.05 compared with the silica group; and & *P* < 0.05 for the silica group compared with the silica plus anti-CD25 group).

Our previous studies showed that Treg was an important subset of regulatory immune cells that influenced the Th response during experimental silicosis in mice^3^,^7^. We evaluated whether Treg was involved in the change of Th responses resulting from insufficient B10. Figure 7 shows that the percentage of Foxp3^+^ Treg significantly decreased in the silica plus anti-CD22 group compared with that of the silica group. To confirm the change of Treg, the level of its typical nuclear transcription factor Foxp3 was examined by real time PCR (Fig. 7c). The expression of Foxp3 was significantly induced by silica instillation, and anti-CD22 treatment limited the increase of Foxp3. This indicated that insufficient B10 restricted Treg induction caused by silica instillation. To determine whether Treg depletion could have a negative regulatory effect on B10, 100 μg of anti-CD25 monoclonal antibody (mAb) was used to deplete Treg during early and late time points (Fig. 7d). We monitored the percentage of B10 in HLN and spleen after Treg depletion during different stages of silica-induced lung inflammation and fibrosis. Flow cytometry data indicated that the percentage of B10 decreased slightly in HLN on day 7, but otherwise no significant changes were observed after Treg depletion during the early inflammatory stage or the late fibrosis stage (Fig. 7e). These data suggested that B10 was not influenced by Treg depletion during silica-induced lung inflammation and fibrosis.

To further evaluate the regulatory mechanism of B10, the major inhibitory cytokine IL-10 was studied by performing real time PCR and CBA analyses. The expression of IL-10 increased gradually after silica instillation, which was consistent with our previous studies. Anti-CD22 treatment led to a considerable reduction in IL-10 expression (Fig. 7f). The secretion of IL-10 also was reduced in the silica plus anti-CD22 group compared with that of the silica group on day 28 after silica instillation (Fig. 7g). These data suggest that insufficient B10 could restrict the level of inhibitory cytokine IL-10.

## Discussion

Studies on regulatory B cells began in the 1970s^32^, and their diverse phenotypes were associated with CD5, CD1d, TIM-1, CD24, and CD38 cell-surface markers in mice and humans^12^,^18^,^33^,^34^. However, none of these markers uniquely defined all IL-10-producing regulatory B cells, which were reported to participate in airway inflammation, allergic disease, and tumorigenesis^35^,^36^. The current study used CD19 and IL-10 to delineate the CD19^+^IL-10^+^ regulatory B cells (B10). The *in vivo* role of B10 has been investigated using anti-CD20 antibody and CD19 knockout mice^37^. IL-10 knockout mice have been used in many studies because B10 is an IL-10-producing regulatory B cell. However, these knockout methods affected other B cell subtypes or other IL-10-producing cells in addition to B10. Recent work showed that anti-CD22 antibody preferentially depleted regulatory B cells in mice, and this approach has been used in numerous animal experiments during the past 5 years^38^,^39^,^40^. Our results also demonstrated that anti-CD22 application could specifically reduce the number of B10 (see Supplementary Figure S1). Therefore, we used anti-CD22 treatment in this study to generate the B10-deficiency mouse model. Figure 1 shows that anti-CD22 treatment attenuated B10 induction after silica instillation in spleen and HLN.

B10 has been characterized as an immune suppressive regulatory cell. B10 reduced the accumulation of white blood cells (WBCs) in ovalbumin (OVA) -immunized mice^41^. Deficiency of B10 could amplify the allergic inflammation^42^. B10 could influence the initiation of inflammation and the development of airway inflammatory disease^39^,^43^. In the current study, insufficient endogenous B10 promoted the accumulation of inflammatory cells, and elevated the secretion of TNF-α and IL-6 in BALF after silica instillation. TNF-α is secreted primarily by macrophages, and the abundant accumulation of macrophages in BALF contributed to the elevated level of TNF-α. The elevated TNF-α could further recruit a large number of inflammatory cells and amplify the early stage of lung inflammation. Recruited inflammatory cells led to a high level of IL-6 secretion, which stimulated the immune response and promoted inflammatory cytokine production. Insufficient B10 amplified lung inflammatory responses, which were accompanied by the appearance of fewer mature silicotic nodules and lower levels of collagen deposition. The classic profibrotic cytokine IL-13 reduced the levels of many inflammatory cytokines and induced TGF-β signaling, which was essential in lung fibrosis. Insufficient B10 attenuated silica-induced IL-13 expression. Low IL-13 levels were inadequate to suppress inflammatory cytokines such as IFN-γ. Reduced IL-13 was unable to promote the expression of TGF-β and its signaling pathway, which might attenuate lung fibrosis. Although B10 was believed to be a subtype of B1 cells, the unique phenotype of B10 in regulating the development of silicosis had not been identified. The principal producers of IL-10 in B cell subtypes might be CD1d^+^CD5^+^ B cells or CD21^+^CD23^+^ B cells, although this requires further study and verification. These combined results indicate that B10 insufficiency regulates the development of lung inflammation and may attenuate silica-induced lung fibrosis.

Although the regulatory mechanism of B10 is still controversial, it is believed that the Th immune response is indispensable^44^,^45^. B10 depletion significantly reduced the proliferation of Ag-specific CD4^+^ T cells and cytokine secretion^46^. Transfer of B10 to IL-10^−/−^ arthritic mice inhibited Th1 and Th17 immune responses^44^,^45^. An *in vitro* study showed that although B10 influenced TNF-α expression and Th1 cells, T cell proliferation was not affected by B10. Our previous study showed that Th1 immune response dominated during the early inflammation stage in an experimental silicosis animal model, whereas Th2 immune response was primarily during the late fibrosis stage. The current study showed that the frequency of Th1 cells in HLN was enhanced by insufficient B10. The typical cytokine IFN-γ and the transcription factor T-bet were elevated to various degrees, indicating that insufficient B10 could exacerbate Th1 immune response. The levels of IL-4 and Th2 transcription factor GATA3 were suppressed especially in the late fibrosis stage, when the mice with developing silicosis already shifted the Th balance to favor Th2 predominance^47^. The restricted Th2 immune response can be partially attributed to the change in the Th1/Th2 balance. B10 also could directly influence the development of Th2 cells^48^. Different studies have reported different roles for B10 in the Th17 immune response^19^. Some studies reported that B10 inhibited Th17 immune response in the absence of Th1^49^. In the current study, IL-1β expression was clearly reduced by anti-CD22 treatment. Both IL-1β and IL-23 promoted Th17 cell differentiation and cytokine secretion by regulating its transcription factor ROR-γt^31^,^50^. IL-23 expression of also was suppressed by anti-CD22 treatment. As a result, the level of the typical Th17 cytokine, IL-17, was reduced. We observed that insufficient B10 reduced the levels of IL-17 and its related cytokines and reduced the frequency of Th17 cells, perhaps because the Th1/Th17 balance largely controls changes in Th17 during the immune response. No matter how complex the change of Th immune response was, insufficient B10 modulated the Th balance toward Th1 predominance after silica instillation.

Next, we investigated how B10 regulated the Th immune response. In general, the regulatory function of B10 might be based on direct secretion of regulatory factors and/or indirect interaction with other regulatory cells. However, the regulatory mechanism of B10 in silica-induced lung inflammation and fibrosis was unclear. Existing evidence indicated that regulatory B cells could modulate the T cell response through different Treg subtypes^51^. An animal study showed that tumor-induced B10 could convert resting CD4^+^ non-Treg into Treg^21^. B10 is reported to increase Treg accumulation and promote Treg function by stimulating Foxp3 and CTLA-4 expression^23^. Conversely, B10 and Treg were reported to act independently in regulating autoimmune disease^39^. The regulatory function of Treg was not affected by the absence of B10 according to study of a multiple sclerosis animal model^22^. In the current study, insufficient B10 reduced the frequency of Treg (Fig. 7), whereas Treg depletion did not influence B10 accumulation at the inflammatory site and in the systemic lymphoid organ. These results indicate that B10 might act earlier than Treg during silica-induced lung inflammation and fibrosis; this conclusion also is supported by other studies^51^.

Previous work showed that Foxp3^+^ Treg regulates the development of silica-induced lung inflammation and fibrosis by modulating the Th immune response. Treg could promote the transition from the Th1 to the Th2 response, either by cell-cell contact or cytokine secretion^3^. Similarly, B10 regulation was based on either cell-cell contact through molecules on the surface or by releasing cytokines^51^. B10 could secrete inhibitory cytokine IL-10 to control the Th immune response. The suppressive function of IL-10 has been shown. The Th17 immune response could be effectively influenced by IL-10^49^, and IL-10 could participate in modulating the Th1/Th2/Th17 microenvironment^52^,^53^. CTLA-4 and GITR are reported to maintain Treg function in an IL-10-independent manner^54^. In this study, insufficient B10 attenuated the increase in IL-10, leading to amplification of the Th1-dominated inflammatory response. Treg reduction resulting from insufficient B10 also could reduce the level of IL-10.

**Figure 8:**
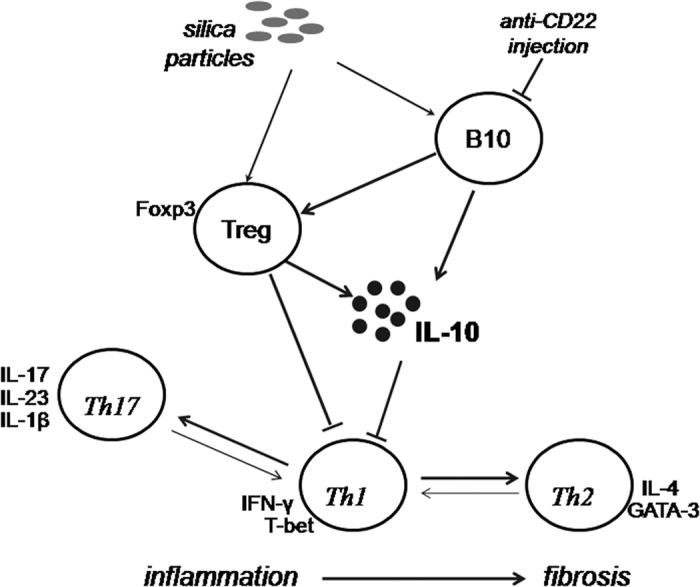
Schematic representation of the role of B10 in regulating Th immune responses during silica-induced lung inflammation and fibrosis.

The combined results of our study show that insufficient B10 could exacerbate silicosis-induced lung inflammation and attenuate lung fibrosis (Fig. 8). Multiple Th immune responses, Th1, Th2, and Th17, were involved in B10 regulation of silica-induced lung inflammation and fibrosis. Insufficient B10 could modulate the Th immune balance toward Th1 predominance. B10 regulation of Th responses might be associated with Treg and inhibitory cytokine IL-10.

## Methods

### Animals

Female C57BL/6 mice were purchased from SLAC Laboratory Animal Co. Ltfd. (Shanghai, China). All animals were housed in a pathogen-free environment and maintained on standard mouse chow at an environmental temperature of 24±1 °C, with 12 h light/12 h dark cycles, and water *ad libitum*. All animal experiments were approved by the Animal Care and Use Committee at the China Medical University (CMU62043018), which complied with the National Institute of Health Guide for the Care and Use of Laboratory Animals. The study was performed in accordance with the approved guidelines.

### Silica exposure

Crystalline silica particles (Min-U-Sil 5 ground silica; size distribution, 97% <5 μm diameter and 80% <3 μm diameter; median diameter 1.4 μm) were purchased from the U.S. Silica Company (Frederick, MD, USA). Silica was ground in saline for 3 h, boiled in 1 N HCl, washed, dried, and suspended in sterile saline. Suspensions were sonicated for 10 min before use. According to their weight, 70 female mice (6–8 weeks old) were randomly allocated into 5 groups as follows: saline group, saline plus anti-CD22 group, silica group, silica plus anti-CD22 group and silica plus anti-CD25 group. Mice were anesthetized with intraperitoneal injection of 2% pentobarbital sodium (45 mg/kg body weight), and received 3 mg/50 μl silica suspension intratracheally to induce experimental silicosis. Control mice received 50 μl sterile saline at the same time.

### B10/Treg depletion

To deplete CD19^+^IL10^+^ regulatory B cells, mice were injected intraperitoneally with 300 μg anti-CD22 antibody (KH2014176, F239, Sangon Biotech, Shanghai, China) one day before silicosis induction, and repeatedly treated i.p. with 300 μg anti-CD22 antibody every 7 days after induction for continuing depletion^40^. To deplete CD4^+^Foxp3^+^ Treg, mice received intraperitoneal injection of 100 μg of anti-CD25 mAb (PC61; BioLegend, San Diego, CA, USA) as described previously^3^. IgG1 was used as control.

### Counting inflammatory cells

Mice were sacrificed on day 7, 28, or 56 after silica exposure. Bronchoalveolar lavage fluid was obtained by cannulating the trachea, removing the lungs, and then injecting and retrieving  ml aliquots of sterile saline 3 times. BALF was centrifuged at 1,500 rpm for 8 min at 4 °C. RBCs were lysed, and the BALF cell pellet was washed and resuspended in phosphate-buffered saline (PBS). The total cell counts were determined using standard hematological procedures. BALF cytospin was prepared and stained using the Wright-Giemsa method. Macrophages, neutrophils, and lymphocytes were identified in a population of 200 cells using standard morphological criteria.

### Pathological examination

Lungs were fixed in 4% paraformaldehyde-PBS. The tissue was embedded in paraffin and cut into 6 μm thick sections. The tissue sections were stained with H&E or Masson and mounted for microscopic examination. The silicotic nodules in lung sections were stained with H&E, evaluated in a blinded fashion, and graded according to established criteria^30^ as follows: 0, no observed silicotic nodules; stage I, cellular nodules; stage II, fibrotic cellular nodules; stage III, cellular fibrotic nodules; and stage IV, fibrotic nodules. Silica-induced fibrotic changes and Masson staining in lung section were evaluated in a blinded fashion and the positive area of collagen deposition in lung was quantified using Image-Pro Plus 6.0 software^55^.

### Cell isolation

The hilar lymph nodes (HLN) were harvested, dissected with dissection needles, and digested with 0.25% trypsin for 5 min at 37 °C. Then, 3% fetal bovine serum in PBS was used to quench the digestion. Samples were centrifuged at 1,500 rpm for 8 min at 4 °C. The HLN cell pellet was washed and resuspended in PBS. The spleen was removed, ground, and mechanically dissociated in cold PBS. After lysis of RBCs, spleen cells were washed and resuspended in PBS.

### Flow cytometry

The HLN and spleen cells were stimulated with Leukocyte Activation Cocktail (BD Pharmingen, San Jose, CA, USA) and LPS 10 μg/ml (Sigma-Aldrich, St.Louis, MO, USA) for 5 h, followed by blocking with purified rat anti-mouse CD16/CD32 (2.4G2, BD Pharmingen) for 10 min at 4 °C. Cell surface staining was performed with PerCP-Cy5.5 conjugated CD4 (RM4-5) or PerCP-Cy5.5 conjugated CD19 (1D3, BD Pharmingen) as described previously^3^. Cells were fixed and permeabilized using a fixation/permeabilization kit (eBioscience, San Diego, CA, USA) or BD Cytofix/Cytoperm™ Fixation/Permeabilization Solution kit (BD Pharmingen) according to the manufacturer’s instructions. Then, cells were stained at 4 °C with Alexa Fluor 488-conjugated IFN-γ (XMG1.2), PE-conjugated IL-17A (TC11-18H10), Alexa Fluor 647-conjugated Foxp3 (MF23), or PE-conjugated IL-10 (JES5-16E3, BD Pharmingen). Stained cells were washed twice and resuspended in 1% paraformaldehyde-PBS. Analysis of cell marker expression was performed using a FACSCanto II system (BD, Franklin Lakes, NJ, USA). Dead cells were gated out depending on forward scattering (FSC) and side scattering (SSC). Cells were analyzed with Diva software.

### Cytomeric bead arrays

Secreted protein levels in BALF were examined by CBA assay using mouse Th1/Th2/Th17 cytokine kit (BD Pharmingen) following the manufacturer’s instructions. Generally, multiple capture beads were mixed together, including TNF-α, IL-6, IFN-γ, IL-4, IL-17, and IL-10. The mixed capture beads were co-cultured with 50 μl BALF supernatant and detection reagent for 2 h. The beads were washed carefully and resuspended. Samples were analyzed by FACSCanto II system (BD). Data were analyzed with FCAP Array software.

### RNA extraction and real time RT-PCR

Total RNA was isolated from lung homogenates using the TRIzol Reagent (Invitrogen, Carlsbad, CA, USA) according to the manufacturer’s instructions. The RNA concentration and the A_260_/A_280_ ratio were determined using a UV spectrophotometer.

Primers and Taqman probes were designed with Primer3 (http://bioinfo.ut.ee/primer3-0.4.0/primer3/), and the sequences were checked by performing a BLAST search (http://blast.ncbi.nlm.nih.gov/Blast.cgi). PrimeScript RT kit (DRR047A, Takara, Japan) and PrimeScript RT PCR kit (DRR096A, Takara, Japan) were used for real time RT-PCR analysis. Briefly, 2 μg of total lung RNA of each animal from each treatment group at each time point was reverse transcribed in a 20 μl reaction using the following program: 37 °C for 15 min and 85 °C for 5 s. Then, 2 μl of cDNA was used for real time PCR analysis in a 25 μl reaction volume. Amplification efficiency differences between target genes and housekeeping genes were identified by comparing the slopes of the standard curves. The PCR reactions were run on an ABI 7500 cycler (Applied Biosystems) using the following program: 95 °C for 30 s; 40 cycles of 95 °C for 5 s and 60 °C for 34 s. Analyses were performed using the 7500 system software (Applied Biosystems).

### Statistical analyses

Data were analyzed for statistical significance using SPSS 19.0 software. The differences between values were evaluated through a one-way analysis of variance (ANOVA) followed by pair-wise comparison with the Student-Newman-Keuls test. *P* < 0.05 was considered statistically significant. All values are means ± SEM.

## Additional Information

**How to cite this article**: Liu, F. *et al*. Role of IL-10-producing regulatory B cells in modulating T-helper cell immune responses during silica-induced lung inflammation and fibrosis. *Sci. Rep.* **6**, 28911; doi: 10.1038/srep28911 (2016).

## Supplementary Material

Supplementary Information
