# Supplementary material for: Role of IL-10-producing regulatory B cells in modulating T-helper cell immune responses during silica-induced lung inflammation and fibrosis
Source: Sci Rep. 2016 Jun 29;6:28911. doi: 10.1038/srep28911 (PMC4926212; doi:10.1038/srep28911)
Supplement: Supplementary Information [file srep28911-s1.pdf]

Role of IL-10-producing regulatory B cells in modulating T-helper cell immune responses during silica-induced lung inflammation and fibrosis

Fangwei Liu[1], Wujing Dai[1], Chao Li[1], Xiaowei Lu[1], Ying Chen[1], Dong Weng[2], Jie Chen[1*]

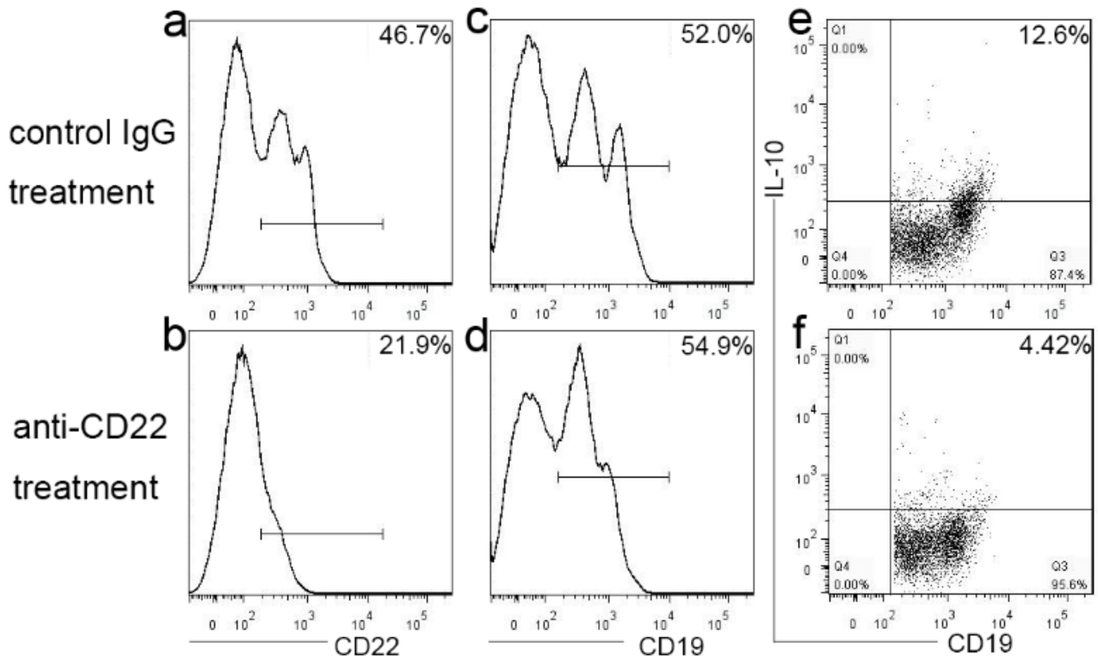

Figure S1. Effect of anti-CD22 treatment on different subsets in spleen. Cells from mice treated with anti-CD22 antibody (b, d, f) and cells from mice treated with control IgG antibody (a, c, e) were stained with anti-CD22, anti-CD19 and anti-IL-10 fluorescent antibodies at day7 after silica instillation. (a, b) The percentage of $CD22^+$ subset was decreased obviously by anti-CD22 treatment. (c, d) The percentage of $CD19^+$ subset was not decreased after anti-CD22 treatment. (e, f) The percentage of $CD19^+IL\text{-}10^+$ subset was decreased by anti-CD22 treatment after silica instillation.

Table S1. Effect of anti-CD22 treatment on the development of silicotic nodules in mice lung

Groups	7 days after instillation		28 days after instillation		56 days after instillation	
	n	silicotic nodule grade	n	silicotic nodule grade	n	silicotic nodule grade
saline	5	0	5	0	5	0
saline+anti-CD22	5	0	5	0	5	0
silica	5	I	5	I^+ - II	5	II^+ - III
silica+anti-CD22	5	I	5	I - I^+	5	I - II
